# Extensive translational regulation during seed germination revealed by polysomal profiling

**DOI:** 10.1111/nph.14355

**Published:** 2016-12-09

**Authors:** Bing Bai, Alessia Peviani, Sjors van der Horst, Magdalena Gamm, ‎Berend Snel, Leónie Bentsink, Johannes Hanson

**Affiliations:** ^1^ Department of Molecular Plant PhysiologyUtrecht University3584 CHUtrechtthe Netherlands; ^2^ Wageningen Seed LaboratoryLaboratory of Plant PhysiologyWageningen University6708 PBWageningenthe Netherlands; ^3^ Theoretical Biology and BioinformaticsUtrecht University3584 CHUtrechtthe Netherlands; ^4^ Umeå Plant Science CentreDepartment of Plant PhysiologyUniversity of UmeåUmeåSE‐901 87Sweden

**Keywords:** Arabidopsis, germination, imbibition, polysomal profiling, ribosome, RNA structure, seedling establishment, translatomics

## Abstract

- This work investigates the extent of translational regulation during seed germination.
- The polysome occupancy of each gene is determined by genome‐wide profiling of total mRNA and polysome‐associated mRNA. This reveals extensive translational regulation during *Arabidopsis thaliana* seed germination.
- The polysome occupancy of thousands of individual mRNAs changes to a large extent during the germination process. Intriguingly, these changes are restricted to two temporal phases (shifts) during germination, seed hydration and germination. Sequence features, such as upstream open reading frame number, transcript length, mRNA stability, secondary structures, and the presence and location of specific motifs correlated with this translational regulation. These features differed significantly between the two shifts, indicating that independent mechanisms regulate translation during seed germination.
- This study reveals substantial translational dynamics during seed germination and identifies development‐dependent sequence features and *cis* elements that correlate with the translation control, uncovering a novel and important layer of gene regulation during seed germination.

## Introduction

Seed germination represents the start of a new plant life cycle. In seed plants, it involves the switch from a quiescent (dry seed) state to a metabolically active embryo which breaks through the encapsulating structures (endosperm and testa) to establish a young seedling. These early stages are critical for plant establishment and crop production. Arabidopsis seed germination is characterized by two visible events. First, the testa (seed coat) ruptures exposing the underlying endosperm layer, and second the endosperm ruptures which occurs when the root tip protrudes through the endosperm thereby completing germination *sensu stricto*.

In Arabidopsis, seed germination is triphasic starting with fast water uptake (imbibition, phase I). Genes encoding ribosomal proteins (r‐proteins) are not transcribed at this developmental stage (Jimenez‐Lopez *et al*., 2011). The first phase ends with a plateau phase (phase II) featuring the activation of a series of metabolic processes facilitating energy production and reserve mobilization. During this process, ribosomal protein gene expression and ribosomal activity increase dramatically, facilitating the *de novo* synthesis of proteins important for seed germination (Fu *et al*., 2005; Dekkers *et al*., 2013; Galland *et al*., 2014). The last phase (III) is characterized by testa and endosperm rupture (germination) followed by radicle extension associated with post‐germination events controlling seed to seedling transition. In the end, germinated seeds are equipped with the machineries and substrates necessary for autotrophic growth (Bewley, 1997). Intensive studies have elucidated the molecular changes during early seed imbibition and seed to seedling transition, including transcriptome (Yu *et al*., 2014), proteome (Gallardo *et al*., 2001) and metabolome (Fait *et al*., 2006) analyses. However, the steady state mRNA pool may not reflect the protein output due to lack of the linearity between transcription and translation (Gibon *et al*., 2006; Baerenfaller *et al*., 2008; Fernie & Stitt, 2012). Polysomal profiling using sucrose gradient‐based fractionation allows the separation of mRNAs based on their association with polysomes and thus the identification of mRNAs actively involved in translation. With high‐throughput mRNA profiling techniques such as microarray analysis and RNA sequencing, thousands of translated mRNAs can be quantified (Mustroph *et al*., 2009b; Layat *et al*., 2014; Lin *et al*., 2014; Vragovic *et al*., 2015). Generating datasets of both the total mRNA and the polysomal bound mRNA allows calculation of the ratio between the abundance of an individual mRNA in the total mRNA fraction and the abundance in the polysomal mRNA fraction. Changes in this ratio between time points or between different treatments indicate changes in the polysome occupancy, showing that a certain mRNA is under translational regulation. Polysome occupancy is partly synonymous with the term polysome loading and is used as a proxy of translational efficiency in the literature. This system has been successfully applied to investigate translational control in, for example, *Saccharomyces cerevisiae* (Arava *et al*., 2003; Halbeisen & Gerber, 2009; Ingolia *et al*., 2009), *Aspergillus fumigatus* (Krishnan *et al*., 2014), a mammalian cell line (de Klerk *et al*., 2015) and *Arabidopsis thaliana* (Jiao & Meyerowitz, 2010; Liu *et al*., 2012, 2013; Juntawong *et al*., 2014; Basbouss‐Serhal *et al*., 2015).

To investigate the degree and dynamics of translational regulation as well as to identify gene sets under translational regulation during germination, total mRNA and polysomal mRNA changes were investigated using microarray analysis of five consecutive stages during Arabidopsis seed germination. Thousands of individual mRNAs whose polysome occupancy was affected were identified. Intriguingly, changes in polysome occupancy were not uniformly present throughout the germination process but were restricted to two temporal phases, one encompassing seed hydration and one seed germination. Using bioinformatic analysis, we were able to correlate the translational regulation to mRNA structure and the presence of sequence motifs present in these mRNAs. Thus, next to the strong transcriptional regulation observed previously during Arabidopsis seed germination, this study identified large sets of genes that are regulated on the translational level, revealing an additional layer of gene expression regulation and its dynamics during germination.

## Materials and Methods

### Plant material and growth conditions

Seeds of the *Arabidopsis thaliana* (L.) Heynh accession Columbia‐0 (Col‐0) were used for all assays described (NASC N60000). The timing of testa and endosperm rupture and seedling greening of fully after‐ripened seeds was determined as described previously (Joosen *et al*., 2010). In brief, two layers of blue blotter paper (Anchor Paper Co., St Paul, MN, USA) were equilibrated with 48 ml of demineralized water in plastic trays (15 × 21 cm). For germination assays, *c*. 50–150 seeds were spread on wetted papers in the germination trays using a mask to ensure accurate spacing. Germination trays were stacked and wrapped in a closed transparent plastic bag to ensure equal illumination from the sides to each plate. The experiment was carried out in a 22°C incubator under continuous light (143 μmol m^−2^ s^−1^). Germination parameters were manually counted.

For ribosome analyses, dry seeds were imbibed as mentioned earlier using three independent biological replicates. Seeds and seedlings were harvested at each physiological state during the seed to seedling transition, frozen in liquid nitrogen followed by freeze‐drying. The dry material was stored at −80°C until further analysed.

### Isolation polysomal mRNA

Polysomal RNA was isolated according to Subramanian (1978) and Mustroph *et al*. (2009a) with some modification. In detail, 400 mg (DW) of freeze‐dried tissue was extracted with 8 ml of polysome extraction buffer (PEB: 400 mM Tris pH 9.0, 0.25 M sucrose, 200 mM KCl, 35 mM MgCl_2_, 5 mM EGTA, 1 mM phenylmethane sulfonyl fluoride, 5 mM dithiothreitol (DTT), 50 μg ml^−1^ cycloheximide, 50 μg ml^−1^ chloramphenicol). The extracts were loaded on top of a sucrose cushion (1.75 M sucrose in PEB) and centrifuged (18 h, 90 000 ***g***) using a Beckman Ti70 rotor for 18 h at 4°C (Beckman Coulter, Brea, CA, USA). The resulting pellet was resuspended in wash buffer (200 mM Tris pH 9.0, 200 mM KCl, 0.025 M EGTA, 35 mM MgCl_2_, 5 mM DTT, 50 μg ml^−1^ cycloheximide, 50 μg ml^−1^ chloramphenicol). Optical density at 260 nm (OD_260_) was measured for the samples which were loaded on a 20–60% linear sucrose gradient, and centrifuged at 190 000 ***g*** for 1.5 h at 4°C using a Beckman SW55 rotor (Beckman Coulter) either according to equal DW or equal OD_260_ values. After ultracentrifugation, the gradients were fractionated into 20 fractions using a Teledyne Isco Density Gradient Fractionation System (Teledyne Isco, Lincoln, NE, USA) with online spectrophotometric detection (at 254 nm). The fractions corresponding to polysomes (mRNAs with two or more ribosomes) were pooled for further analysis. This was performed using at least three independent biological repeats.

The ribosome abundance is reflected by the total area under the curve and was calculated after subtracting the baseline obtained by measuring a blank gradient and normalizing to total area under the curve to account for possible uneven amount of plant material in the samples. Polysomal loading of samples was calculated by comparing the area corresponding to two or more ribosomes (polysomes) after background subtraction and normalization with the total area under the curve.

### Isolation and analysis of RNA species

The relative ratio of ribosomal types was calculated by determining the relative amounts of the small subunits of cytosolic, plastidic and mitochondrial rRNAs by quantitative real‐time PCR (qRT‐PCR) normalized to the geometric mean of the spike‐in standards assuming no presence of naked rRNA species (Piques *et al*., 2009). Aliquots of 300 μl total extract and 800 μl pooled polysome fraction were spiked with a mix of the four eukaryotic poly(A) RNAs including *lys*,* phe*,* thr* and *dap* (Affymetrix, Santa Clara, CA, USA; Ambion, P/N900433), with relative final concentrations of 1 : 100 000, 1 : 50 000, 1 : 25 000 and 1 : 6667, respectively, and purified with TriPure Isolation Reagent (Roche, Basel, Switzerland), followed by clean‐up using RNeasy Mini spin columns (Qiagen, Hilden, Germany) and dissolved in RNase‐free H_2_O. cDNA was synthesized using the iScript™ cDNA synthesis kit (Bio‐Rad, Hercules, CA, USA) according to the manufacturer's protocol. After DNase1 treatment (Thermo Scientific, Waltham, MA, USA), qRT‐PCR was performed using Power SYBR Green (Applied Biosystems, Waltham MA, USA) in a 5 μl reaction using the standard program of the ViiA™ 7 instrument (Applied Biosystems). Data were analysed using ViiA™ 7 Software v.1.1 (Applied BioSystems). Primer amplification efficiency was calculated using linregPCR (Ruijter *et al*., 2009). The quantification of the poly(A) RNA control spikes were used to normalize qRT‐PCR data. All primers used are provided in the Supporting Information, Table S1(a).

### Data analysis

Affymetrix Arabidopsis Gene 1.1 ST Arrays (Affymetrix) were hybridized using the GeneChip^®^ 3′ IVT Express kit (cat. no. 901229; Affymetrix, Santa Clara,CA, USA) according to instructions from the manufacturer. Hybridization data were analysed and gene‐specific signal intensities were computed using the R statistical programming environment (http://www.R-project.com), the BioConductor package affy (Gautier *et al*., 2004) and the Brainarray cdf file v.17.1.0 (http://brainarray.mbni.med.umich.edu/). DNA microarray data are available in the Gene Expression Omnibus (GEO) repository (http://www.ncbi.nlm.nih.gov/geo/) under accession number GSE65780. The limma and affy packages were used for RMA normalization (Irizarry *et al*., 2003). Probe set intensity signals that never exceeded the noise threshold (log_2_Exprs ≤ 4 in all samples) were removed. A linear model and empirical Bayes methods were applied for assessing differential expression (Smyth, 2004) and genes are only considered as differentially expressed when log_2_ fold change (FC) > 1 and *P* < 0.05 adjusted by the false discovery rate (FDR) according to the method of Benjamini & Hochberg (1995). Correlation between RMA normalized biological replicates averaged 0.98 (Pearson's correlation) and ranged between 0.96 and 0.99 (Fig. S1f). Relative RNA levels were validated with qRT‐PCR experiments with four spikes as internal standard for the normalization (Vandesompele *et al*., 2002). DNA sequences and efficiencies of primer pairs used for qRT‐PCR experiments and comparison of relative mRNA levels determined in GeneChip and qRT‐PCR experiments are given in Table S1(a). Principal component analysis (PCA) was performed using TM4 (Saeed *et al*., 2003). Polysome occupancy for each mRNA was calculated by comparison of normalized levels from the polysomal and total RNA. The same criterion for a significant change was used for polysome occupancy as for changes in total and polysomal levels.

### Analysis of identified mRNA sequences

gene trail (http://genetrail.bioinf.uni-sb.de/) and revigo (http://revigo.irb.hr/) were used for over‐representation analysis using default parameters to characterize the dominant transcriptional and translational processes related to seed germination. Geneset enrichment analysis was performed via agriGO (http://bioinfo.cau.edu.cn/agriGO/; TAIR10) using a Fisher test followed by the Hochberg method at a significance level of 0.05. The generated GO lists related to biological processes with a *P*‐value for FDR were put into Revigo with a dispensability cut‐off of 0.05 to remove GO term redundancy.

### Sequence feature analysis

Genes with significantly increased and decreased polysome occupancy at each translational shift were compared with the microarray background that represents total gene sets on the microarray for several sequence features using custom scripts. The distributions of sequence lengths and GC content were evaluated separately for coding DNA sequence (CDS), 5′ untranslated region (5′UTR), 3′UTR and full transcript. CDSs were also analysed for GC levels in third positions of codons (GC3), after removing sequences missing the start codon and/or containing premature stop codons; CDSs shorter than 100 codons were further removed for the codon bias analysis, measured using the effective number of codons (*N*_c_) index (Sun *et al*., 2013). The same analyses were performed separately for the CDSs of protein‐coding genes having both or no annotated UTR (UTRs called present when having length > 1 nucleotide). Given the nonnormality of the distributions of values, a Wilcoxon signed‐rank test was adopted for all statistical comparisons (median as the test statistic).

### RNA structural analysis

Experimentally determined structure scores per nucleotide, as provided by Li *et al*. (2012), were used to calculate average structure scores of the genes with significantly increased and decreased ribosomal association at each developmental switch. Relative scaling was achieved by averaging the structure scores per region (5′UTR, CDS and 3′UTR) in 100 bins. Standard errors were determined and Student's *t*‐tests were performed using the python scipy module (http://www.scipy.org/).

### Motif analysis

DNA motif analyses were performed using the MEME suite (Bailey *et al*., 2009), for full transcript, 5′UTR, CDS and 3′UTR sequences, extracted from the TAIR10 database (http://www.arabidopsis.org/). The minimum and maximum motif width was set to 6 and 10, respectively. If a gene had multiple isoforms, only the TAIR10 representative splice form was used. Background dinucleotide frequencies were provided separately for each sequence region of the transcript. To test the specificity of the resulting motifs, FIMO (Bailey *et al*., 2009) was used to scan all genes represented on the microarray for motif hits in the corresponding sequence type. Motifs with a *P*‐value ≤ 0.001 were considered significant hits. Obtained motif counts were used to compute the enrichment *P*‐value for the gene lists vs the background by means of a one‐tailed Fisher's exact test, performed with a custom script and the R software package (http://www.r-project.org/). For each motif, the positions on the transcripts, as provided by the FIMO output, were used to calculate the relative number of motifs per (relative) position along the mRNA. Relative scaling was performed in a similar fashion as for the structure scores.

## Results

### Translational activation precedes ribosome biogenesis during Arabidopsis seed germination

**Figure 1:**
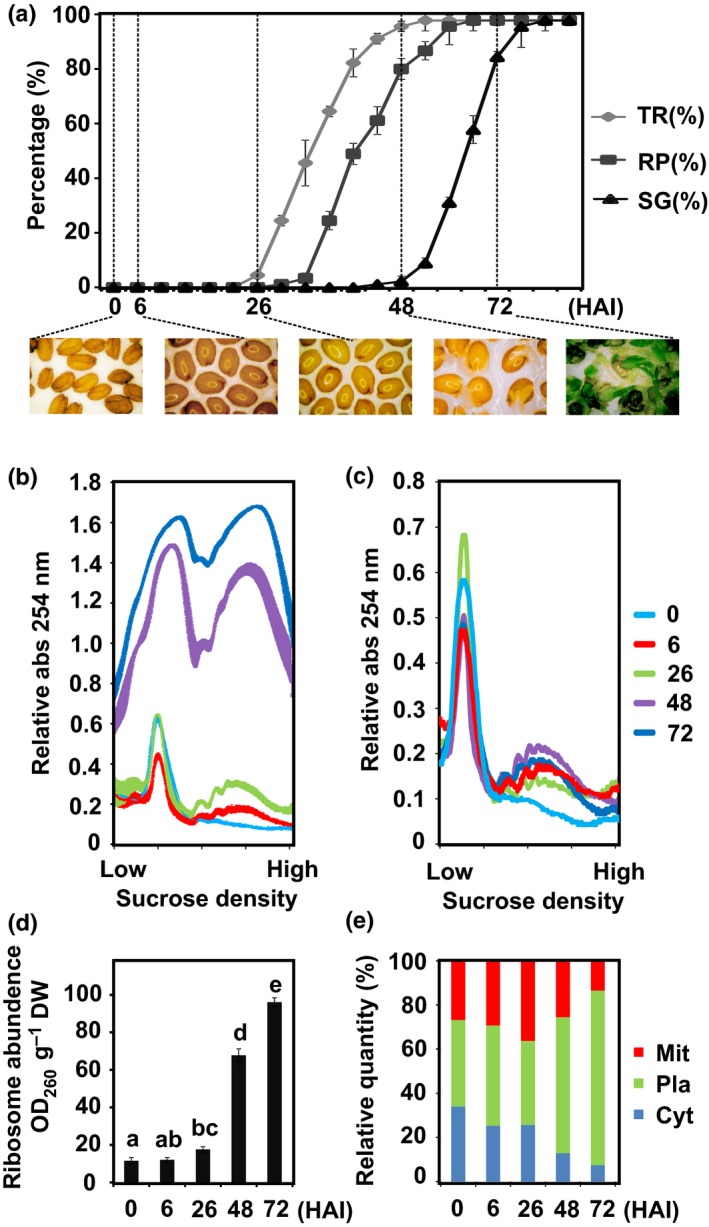
Polysome profiling during seed germination. (a) Frequency of testa rupture (TR), radicle protrusion (RP) and seedling greening (SG) during the Arabidopsis Col‐0 seed to seedling transition. Time points during seed germination include dry seeds, 6, 26 (TR initiation), 48 (80% RP) and 72 h after the start of imbibition (HAI) (80% GS). Data are presented as mean ± SD of three independent replicates. (b) Absorbance profiles of sucrose density gradient fractionated ribosomes for the five time points during the seed to seedling transition. Data are presented as mean ± SD of three independent replicates. SD is indicated by the width of the line. Gradient loading according to equal dry weight. (c) Representative absorbance profiles of sucrose density gradient fractionated ribosomes for the five time points during the seed to seedling transition; gradient loading according to identical RNA loading. (d) Ribosome abundance for the five time points during the seed to seedling transition. Data are presented as mean ± SD of three independent replicates and the letters above each bar indicate the significance (*P* < 0.05). (e) Relative abundance of different ribosomes (Cyt, cytosolic; Pla, plastidic; Mit, mitochondrial).

Monitoring the seed to seedling transition of fully after‐ripened Arabidopsis seeds was performed by scoring the percentage of testa rupture (TR), radicle protrusion (RP) and seedling greening (SG) over time. TR started *c*. 26 h after the start of imbibition (HAI). RP was first observed 35 HAI, and at 48 HAI 80% of the seeds showed radicle protrusion. By 72 HAI, 80% of the seedlings had reached the SG stage and at 82 HAI all the seedlings had turned green (Fig. 1a). The time points (0, 6, 26, 48 and 72 HAI) that mark different physiological stages (dry seeds, early imbibition, and initiation of TR, 80% RP and 80% SG, respectively) were selected for polysome profiling based on both equal DW (Fig. 1b) and equal RNA level in the samples (Fig. 1c). Ribosome profiles changed dramatically during the seed to seedling transition. In dry seeds, ribosomes were mainly present in the monosome form (Fig. 1b,c). Following imbibition ribosome profiles changed. This was first visible by an increase in the polysome peak from dry to 6 HAI (Fig. 1b) concurrent with a decrease of the monosome peak, followed by an increased total area that represents the increase in ribosome abundance (Fig. 1b–d). These newly synthesized ribosomes mostly represent organellar ribosomes, especially plastid ribosomes after 48 HAI, as shown by the relative quantity of ribosomal RNA specific to each organelle (Fig. 1e).

### Transcriptional changes are reflected in polysomal mRNA levels during seed germination

**Figure 2:**
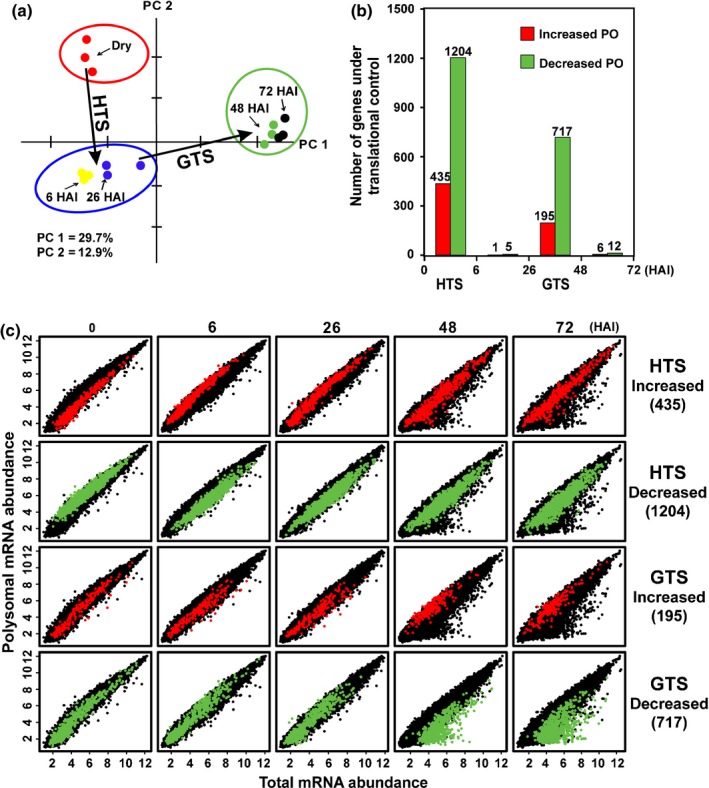
Arabidopsis seed germination is characterized by two translational shifts. (a) Principal component (PC) analysis of polysome occupancy (PO) changes (polysomal mRNA levels/total mRNA levels) during seed germination. The first two components (PC1 and PC2) explain 30 and 13% of the total variation, respectively. (b) The number of mRNAs with changed PO at the two translational shifts, at 6 h after the start of imbibition (HAI) the hydration translational shift (HTS) and 48 HAI, the germination translational shift (GTS). (c) Dynamics of genes underlying the two translational shifts. The levels of total (*x*‐axis) and polysomal (*y*‐axis) mRNAs following seed imbibition (dry seeds, 6, 26, 48 and 72 HAI) are plotted (black). Genes identified with increased (red dots) or decreased (green dots) polysome occupancy are indicated for both the HTS (upper panels) and the GTS (lower panels).

Total mRNA (T) and polysomal mRNA (P) expression levels were analysed to investigate the translational dynamics during the seed to seedling transition using the high‐throughput Gene ST1.1 Array. No obvious RNA degradation was observed in any of the samples and the sample preparation was robust based on the same signal distribution after normalization and the high similarity between sample replicates (*r *>* *0.96 and *r *=* *0.98 on average, Fig. S1). The 19 781 genes with intensities that passed the noise filter (log_2_Exprs > 4) based on the intensity distribution (Fig. S1a,b) in all three independent biological replicate samples for at least one time point during seed germination were subjected to further analysis (Table S1b). The change in mRNA abundance between each stage was determined by comparing RNA levels to the preceding stage (Fig. S2a) as well as to the dry state (Fig. S2b) during seed germination. mRNA levels changed similarly in the total RNA and polysomal RNA (Figs S2, S3). Similar GO functions were over‐represented for both RNA preparations (Fig. S4). The changes at the transcriptional level are in agreement (≥ 50% overlap, FC = 2, FDR = 0.05) with recent data describing transcriptional changes during the seed to seedling transition (Silva *et al*., 2016; Table S1c). The upregulated genes were over‐representing processes involved in protein localization, oxygen and reactive oxygen species metabolic process, ribosome biogenesis, translation, stress responses, cell wall organization, photosynthesis, and lipid transport and localization (Fig. S4b). By contrast, chitin response, abscisic acid response, defence, secondary metabolism, seed development, RNA processing and ribosome biogenesis (both cytosolic and organellar) were over‐represented in the downregulated gene set (Fig. S4c). The genes encoding ribosomal protein genes were strikingly differentially expressed during the seed to seedling transition (Fig. S5). Generally, a strong correlation between transcription and translation across seed germination was observed (Figs 2, S3).

### Polysomal profiling reveals two phases of translational control

To identify genes that are under translational control, we assessed the polysome occupancy of each mRNA species, which is defined as the ratio between the mRNA in the polysome pool and the total mRNA (Bailey‐Serres, 1999; Branco‐Price *et al*., 2005, 2008; Gamm *et al*., 2014). By comparing the polysome occupancy between each stage and the preceding time point, we identified two temporal phases with extensive changes in translational control: between dry seeds and 6 HAI seeds and between 26 and 48 HAI seeds (Fig. 2). Changes exceeding twofold and associated with a corrected *P*‐value of 0.05 were considered significant. We refer to these phases as the hydration and the germination translational shifts (HTS and GTS). In total 1204 genes were downregulated in the HTS (HTS down) and 435 genes were upregulated (HTS up). For the GTS the numbers were 717 (GTS down) and 195 (GTS up). Minor significant translational changes were identified between 6 and 26 HAI and between 48 and 72 HAI (Fig. 2b). The genes identified in two translational shifts during seed germination were largely non‐redundant (Fig. S6a). There was a large overlap with genes subjected to translational control under hypoxia stress (Fig. S6b,c,h), but no significant overlap with genes under translational regulation in the sucrose feeding and starvation experiments or seed dormancy (Fig. S6e–g).

### Dynamics of genes under translational control

**Figure 3:**
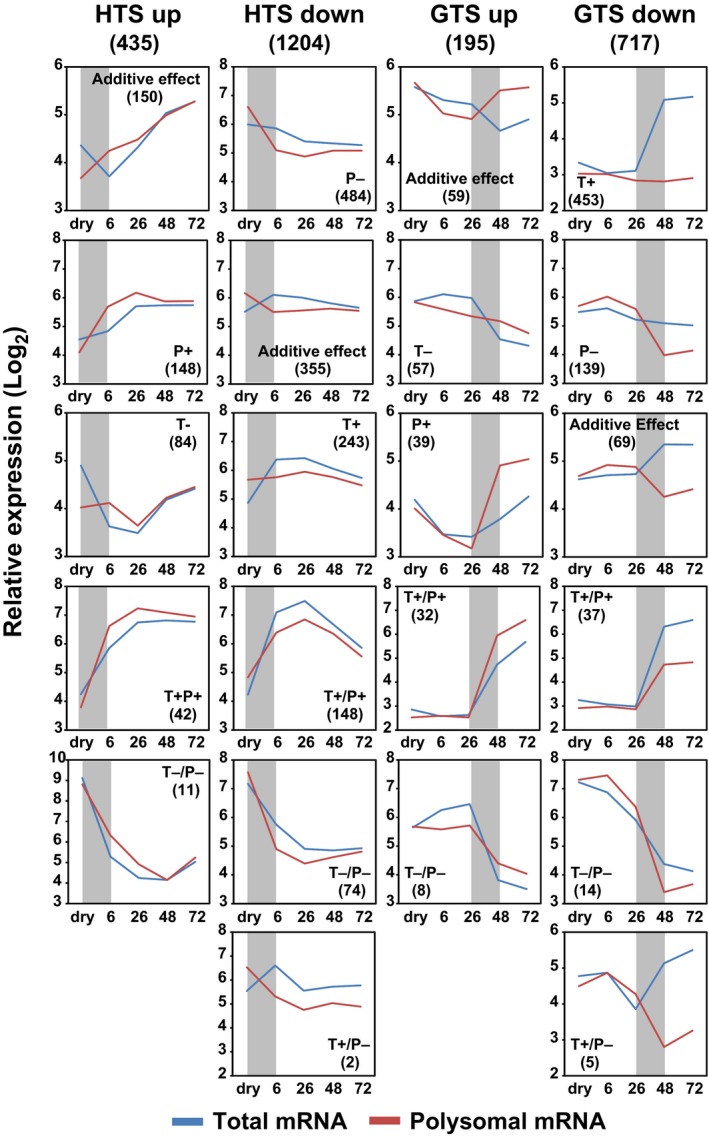
Total RNA and polysomal RNA profile of translational regulated genes during the seed to seedling transition in Arabidopsis. Translational regulated genes are divided into subgroups based on their total RNA and polysomal RNA changes. In total, 22 subgroups under nine categories are identified with their corresponding expression profiles following seed germination and genes numbers under each subgroup in parentheses. T+ represents that the transcription is increased while the translation is unchanged under a specific shift. P+ indicates translation is increased while the total mRNA abundance is unchanged. T+/P+ indicates both transcription and translation are enhanced and an additive effect means that the change of gene expression in either transcription or translation is not significant while the additive effect of the two contributes to the significant change of polysome occupancy. The opposite effect is represented by T−, P− and T−/P− in the corresponding subgroups. HTS, hydration translational shift; GTS, germination translational shift; HAI, hours after the start of imbibition.

To visualize the transcriptional and translational dynamics of genes under translational control, the four different gene sets were highlighted in correlation plots of the different time points either by transcript abundance or by fold change (Figs 2c, S3). This showed that the translationally upregulated genes in HTS were relatively lowly expressed and similarly weakly associated to the polysomes in dry seeds. At 6 HAI these genes were associated with polysomes at higher levels than expected based on their expression, followed by similar levels in both pools during the later imbibition phases. The opposite pattern was shown for the downregulated genes. These genes were generally highly associated with the polysome in dry seeds, and decreased in polysome association at 6 HAI. The translationally upregulated genes in the GTS up gene set were specifically highly associated with the polysomes at 48 HAI and this continues in the later time point, while the downregulated genes were represented by mRNAs associated with polysomes at levels corresponding to total mRNA levels at early time points but specifically less associated with polysomes at the two later time points. In principle, changes in polysomal occupancy can be caused both by changes in the transcript level and by changed association of the mRNA to ribosomes (or combinations thereof). By comparing these effects separately, different patterns emerge. The group of genes downregulated in the HTS seem primarily affected negatively on the polysomal mRNA level, while most of the genes downregulated in GTS down are characterized by a dramatic upregulation on the total mRNA level (Fig. 3). The different patterns between the downregulated genes in the two shifts indicate different regulatory mechanisms at different stages of seed germination.

Examples of genes upregulated in the HTS are the wounding responsive gene (AT4G10270, log_2_ FC = 2.6, FDR = 0.05), HSP‐20 LIKE chaperon super family protein gene (AT4G21870) and glycine rich protein gene (AT3G29075, log_2_ FC = 2.3, FDR = 0.05; Table S1d). The first of these was also reported as highly translationally repressed in response to hypoxia (Branco‐Price *et al*., 2005). By contrast, a dramatic translational reduction was observed for a pyruvate kinase family protein gene (AT3G04050, log_2_ FC = −2.1, FDR = 0.05) while no significant change at total mRNA level was detected. This gene product is important for the conversion of photosynthates into oil in the developing seeds (Andre *et al*., 2007), which is an essential developmental programme during seed maturation. At the GTS, translation of 39 and 139 transcripts specifically increased or decreased independent of transcription (Fig. 3; Table S1d). Representatives of these genes include *CYCLIN‐DEPENDENT KINASE B2* (AT1G20930, log_2_ FC = 2.4, FDR = 0.05), RmlC‐like cupins superfamily protein gene (AT2G18540, log_2_ FC = −2.9, FDR = 0.05) and *WUSHEL RELATED HOMEOBOX2* (AT5G59340, log_2_ FC = −2.4, FDR = 0.05). Furthermore, genes related to seed development such as embryo development genes, seed storage protein genes, late embryogenesis abundant genes, stress response genes such as heat shock protein genes, hormone related abscisic acid (ABA) and auxin response genes, metabolic genes related to lipid and sucrose metabolism, cell wall related genes, chloroplast related genes and ribosomal protein genes were identified as dominant gene groups that were under intensive translational control (Table S1e,f).

### Transcript features correlate with translational regulation

**Figure 4:**
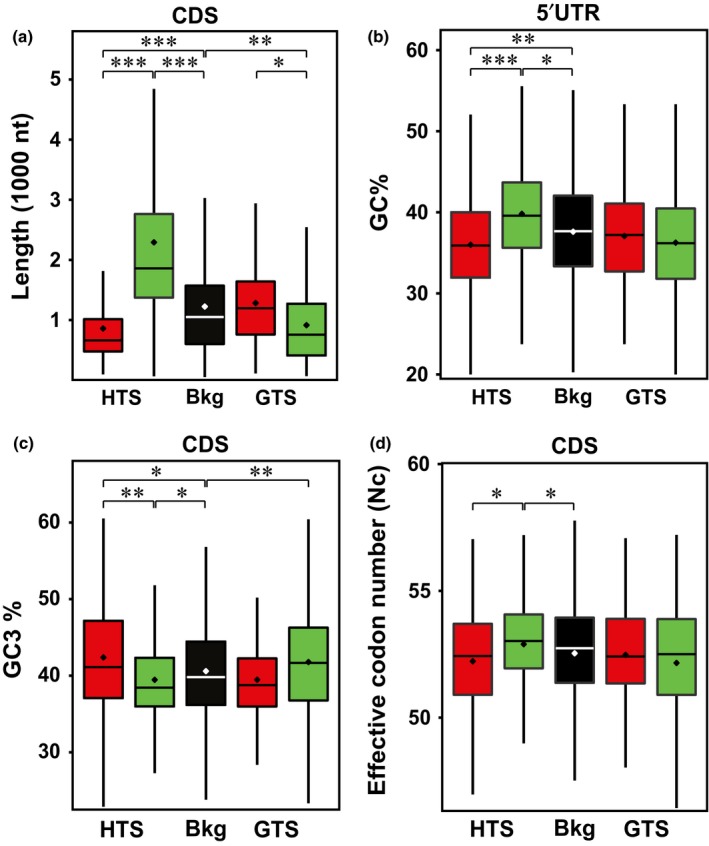
mRNAs of the two shifts during the seed to seedling transition in Arabidopsis are characterized by distinct sequence features. (a) Length of coding DNA sequence (CDS), (b) GC content of 5′UTR, (c) GC3 content and (d) effective codon number (*N*_c_) of the CDS are shown. Colour scheme: black, microarray background (Bkg); red or green, translationally up‐ or downregulated genes at the hydration translational shift (HTS) and germination translational shift (GTS), respectively. Error bars represent SE (*, *P* < E‐10; **, *P* < E‐20; ***, *P* < E‐50). *P*‐values are calculated by a Wilcoxon nonparametric test.

To investigate whether transcript features correlate with translational regulation, we determined transcript length and GC content of the translationally regulated mRNAs. It is established that short transcripts and transcripts with low GC content are in general more efficiently translated than long transcripts (Qu *et al*., 2011; Valleriani *et al*., 2011; Liu *et al*., 2012). For the HTS we found significantly longer genes in the downregulated set compared with the upregulated and background gene sets (all genes present on the array). However, the GTS showed an opposite pattern (Figs 4a, S7; Table S1g). This indicates that translation at the shifts is regulated by distinct mechanisms. Significantly higher GC contents were identified in the 5′UTR and 3′UTR of the HTS and in the CDS of the GTS downregulated genes, which correlates with suppression of translation (as determined by changed polysome occupancy). Due to redundancy in the genetic code, most amino acids are encoded by several synonymous codons, although it is thought that some codons are translated more efficiently than others. This codon bias is calculated based on the effective number of codons (*N*_c_), the number of codons used in a gene, ranging from 20 (extreme bias) to 61 (all codons used; Hershberg & Petrov, 2008, 2009). Codon degeneracy is nearly completely related to the third‐base position (Crick, 1966) and related to the GC content at this position. Interestingly, polysome occupancy changes during seed hydration correlate negatively with *N*_c_ and positively with GC3 (guanine and cytosine content in the third codon position; synonymous sites) (Figs 4, S7). Highly translated transcripts often use the optimal codons, as observed in several species (Ikemura, 1985; Bulmer, 1987; Akashi, 1994; Duret, 2000; Drummond & Wilke, 2008; Shabalina *et al*., 2013).

Upstream open reading frames (uORFs) are commonly involved in regulating translation. We tested whether genes with uORFs are over‐represented in the translationally regulated gene list during seed germination by a χ^2^‐test, using the published 2020 uORF containing genes identified in the Arabidopsis genome (Juntawong *et al.,* 2014). At the HTS, 15 out of 435 genes were identified to harbour uORFs in the translationally upregulated genes, significantly lower than the uORF occurrence in the genome (expected = 31.32, *P* < 0.005); by contrast, significantly more genes (110 out of 1204) with uORFs (expected = 75.60, *P* < 0.005) were present in the downregulated genes. For the GTS there was no enrichment for uORFs in the translationally upregulated genes (20/195) while a significantly low occurrence was detected for the downregulated genes (23/717) (expected = 51.32, *P* < 0.005).

We mapped the genes shown to be translationally regulated to the dataset of mRNA decay profiles and associated sequence features of mRNAs of cultured cells (Narsai *et al*., 2007). The genes translationally regulated in the HTS differed significantly in transcript stability. Translationally downregulated genes of the shift were significantly less stable than expected by random and the upregulated genes were more stable as judged from the mRNA stability measurements in Arabidopsis cell culture. There were significantly high numbers of introns especially in the CDS and 3′UTR, while other mRNA characteristics of the genes were not different from expected values (Table S1h).

**Figure 5:**
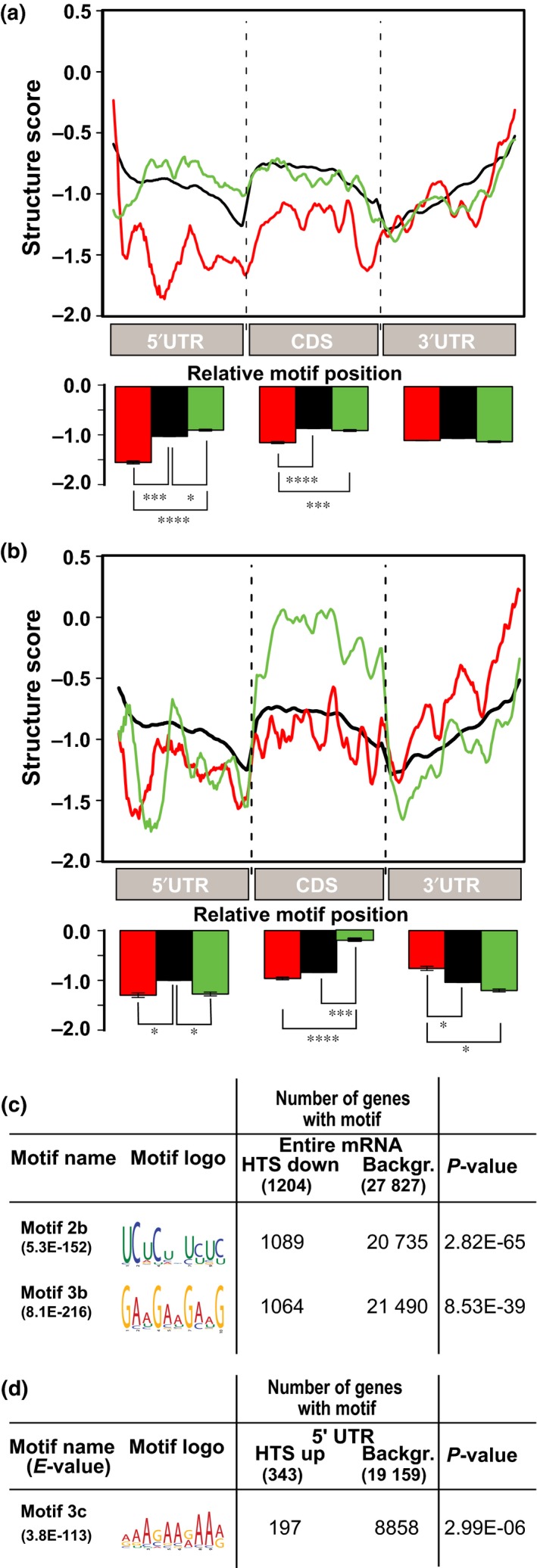
Secondary structures and motif features correlate with polysome association. The average structure score is plotted over the 5′UTR, coding DNA sequence (CDS) and 3′UTR of the (a) hydration translational shift (HTS) and (b) germination translational shift (GTS) affected transcripts. Background: all transcripts on the array (black line), translational up (red line) or translational down (green line). *P*‐values are calculated by a Student's test according to Li *et al*. (2012). The bar plots represent the mean structure scores per transcript region (5′UTR, CDS and 3′UTR, respectively). Error bars represent SE (*, *P* < E‐10; ***, *P* < E‐50; ****, *P* < E‐100 ). Significantly enriched motifs are detected across (c) the whole transcript length (cDNA) and (d) 5′UTR. *P*‐values are calculated by a Fisher's *t*‐test. Note: several sequence motifs overlapping with motif 2b were identified. Only the most significant is depicted.

The role of the mRNA's secondary structure on translational control was tested by mapping the translationally controlled genes to the experimentally derived structural score defined by Li *et al*. (2012). This score is an indicator of transcript complexity, in which high structure scores are equivalent to more double‐stranded (ds) than single‐stranded (ss) RNA at a certain position in a transcript and vice versa for low structure scores. Average structure scores were plotted over the 5′UTR, CDS and 3′UTR for both translational shifts (Fig. 5a,b) and compared with the average score of all genes present on the array (background). In general, mRNAs *in vitro* have been shown to have a steep decrease in structure at the start and stop codon, which is a conserved structural feature for eukaryotes facilitating accessibility of the ribosome for translational initiation (Kozak, 2005; Kertesz *et al*., 2010; Li *et al*., 2012). At the HTS, upregulated mRNAs are less structured in the 5′UTR and CDS than those downregulated, which suggests that mRNAs with lower structure scores are translationally favoured over more structured transcripts at specific stages. The GTS downregulated mRNAs have an overall higher structure score in the CDS, and the opposite trend was found in the 3′UTR. The high structure of the CDS may attenuate the progression of the ribosomes and thereby inhibit translation of these mRNAs at later stages.

Motif analysis on four regions (5′UTR, CDS, 3′UTR and the whole transcript) for both shifts revealed three significantly (*P* < E‐5) enriched motifs (Figs 5, S8), all for the HTS. One was present in the 5′UTR of the HTS up transcripts, and two in the entire transcript RNA sequence of the HTS down set.

Motif 3c was significantly enriched in the 5′UTR of translationally enhanced transcripts in the HTS. This adenosine‐enriched tract is mainly localized in the 50 nucleotide region upstream of the start codon which could potentially bind to translation initiation factors and enhance translation initiation (Fig. S8) (Xia *et al*., 2011).

## Discussion

**Figure 6:**
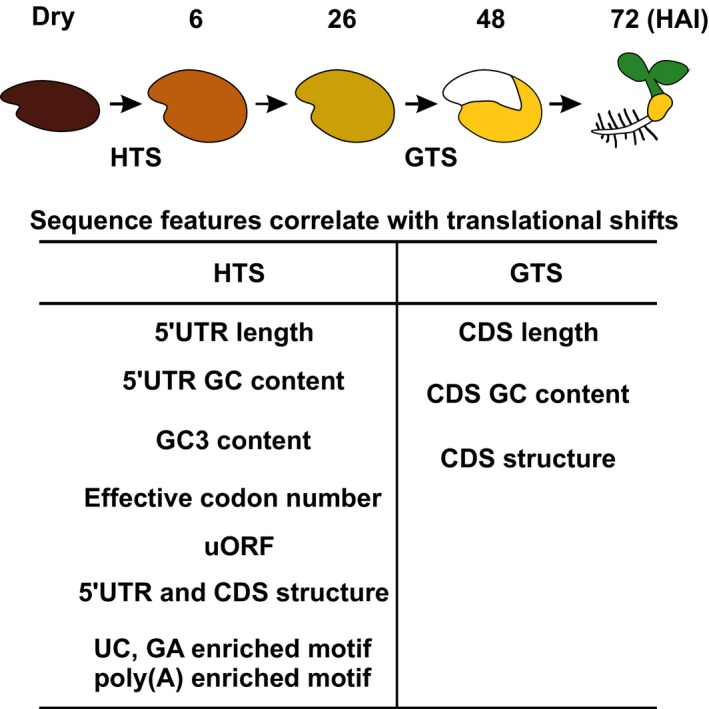
Summary of the sequence features in mRNAs identified as translationally regulated during germination. The upper panel shows a schematic presentation of the transition from a dry seed to a seedling at 72 h after the start of imbibition (HAI). The two phases where large sets of mRNAs are under translational control, the hydration translational shift (HTS) and germination translational shift (GTS), are indicated. The identified features that correlate with these translational shifts are listed. CDS, coding DNA sequence; uORF, upstream open reading frame; UC, pyrimidine; GA, purine.

Translation is essential for seed germination and apparently mRNAs stored in the dry seed are translated during germination (Rajjou *et al*., 2004; Narsai *et al*., 2011; Galland *et al*., 2014; Layat *et al*., 2014). However, an accurate temporal evaluation of the extent of translational regulation during seed germination is missing. Here we present a time course study on the translational dynamics, which reveals that translational activation precedes ribosome biogenesis during the seed to seedling transition. Polysome profiling identified thousands of genes differentially transcribed and translated with temporal resolution. Thus, polysome profiling efficiently helps in identifying genes regulated during seed germination. Our data demonstrated a large overlap of differentially regulated genes on both the transcriptional and the translational level, a natural consequence of mRNA‐dependent translation (Figs S2a, S3). However, by analysing polysome occupancy, defined as the ratio of polysome‐associated and total mRNA levels, across the course of germination we found two phases where the polysome occupancy of thousands of mRNAs changes. This extensive translational regulation during seed germination exceeds what has been shown for translational regulation in other studies both in the numbers of affected genes and in the extent of the effect (Nicolai *et al*., 2006; Gamm *et al*., 2014; Lin *et al*., 2014). These two major shifts are here referred to as the HTS and GTS (Fig. 6). The shifts occur in temporal correlation with key stages of the seed to seedling transition and might refer to physiological control points (Fig. 6), and distinct genes are affected in the two shifts (Fig. S5a). Thus, translational control during seed germination is stage specific and possibly represents different development‐dependent regulatory mechanisms as revealed by the different secondary structures and motifs identified in the two shifts. (Fig. S6b–f; Nicolai *et al*., 2006; Branco‐Price *et al*., 2008; Gamm *et al*., 2014; Juntawong *et al*., 2014; Lin *et al*., 2014; Sorenson & Bailey‐Serres, 2014). Overlap is the largest with translationally regulated genes under hypoxia stress (*c*. 25% of the total identified genes under translational control during seed germination). This may be related to low oxygen responses during the seed to seedling transition.

We have identified sequence features that correlate with translational regulation (Fig. 4). Different features correlate with different translational shifts. For the HTS we found that a reduced number of uORFs, transcript length, GC content and secondary structure correlate with an upregulation of translation. The translationally regulated genes differ in secondary structure, as judged by comparison to the *in vitro* structure of the mRNAs (Fig. 4a,b). The different level of secondary structure of the translationally regulated mRNAs indicates the possibility that structural features are important for this regulation. Since the translationally regulated transcripts identified differ between the two shifts, there should be additional factors that affect the translational regulation. One can envisage a model in which sensitivity to structure at different stages of the seed to seedling transition is mediated by differential activity of RNA helicases, which are dedicated to unpacking the annealed nucleic acid strands such as secondary structures of the RNA complex. Whether helicases play a role in translational regulation of the two shifts remains to be investigated. Other factors that might affect translation are RNA binding proteins specifically interacting with the identified elements in translationally regulated mRNAs. The Arabidopsis genome encodes hundreds of RNA binding proteins and for one class, PUF (proteins that are characterized by the presence of a conserved Pumilio homology domain) proteins, a role in germination has recently been proposed (Xiang *et al*., 2014). The pyrimidine (UC) and purine (GA) enriched motifs identified among the transcripts in the HTS may represent binding sites for polypyrimidine tract‐binding protein, PTB (Singh *et al*., 1995; Perez *et al*., 1997; Oberstrass *et al*., 2005). Motif 3b GAAGAAGAAG is similar to the target sequence (GAAGAAGAAGCUC) of SERINE/ARGININE‐RICH PROTEIN SPLICING FACTOR 40, which acts as an exon enhancer mediated by a complex of nuclear proteins (Yeakley *et al*., 1996). The Arabidopsis SR paralogue SERINE/ARGININE‐RICH SC35‐LIKE SPLICING FACTOR 33 has been identified and plays a role in regulating alternative splicing (Thomas *et al*., 2012). Although not investigated here, splicing might play a role in the HTS as introns are specifically more frequent in HTS mRNAs (Table S1h). Interestingly, the ribosomal protein genes are differentially expressed during the seed to seedling transition (Fig. S5). This may be affecting the composition of the translating ribosomes and thereby the selection of translated mRNAs.

Overall, our data reveal a model of changing translational regulation during seed germination and seedling establishment (Fig. 6). The extensive translational regulation during germination and the changes therein are unlikely to be regulated by a single mechanism. The diversity of sequence features identified favours a multifactorial model. Further research will focus on how these identified features are recognized and thus mediate the translation control. The Arabidopsis genome harbours hundreds of mRNA binding proteins, of which a large majority have no assigned function. The regulators of translation during seed germination are likely to be found in this group of interesting proteins.

## Author contributions

B.B., J.H. and L.B. designed the experiments. B.B. performed the ribosome fractionation and conducted the gene expression microarray experiments. B.B., L.B., J.H., A.P., M.G., S.v.d.H. and B.S. directed the design of analysis approaches. B.B., A.P. and S.v.d.H. conducted the analyses. B.B., L.B. and J.H. drafted the manuscript. All authors participated in revising and editing the manuscript.

## Supporting information

Please note: Wiley Blackwell are not responsible for the content or functionality of any Supporting Information supplied by the authors. Any queries (other than missing material) should be directed to the *New Phytologist* Central Office.

**Fig. S1** Gene 1.1 ST GeneChip quality assessment and reproducibility.
**Fig. S2** Coordinated transcriptional and translational expression changes during the seed to seedling transition.
**Fig. S3** Arabidopsis seed to seedling transition is characterized by two translational shifts.
**Fig. S4** Temporal functional changes of total RNA and polysomal RNA during the seed to seedling transition using over‐representation analysis.
**Fig. S5** Ribosomal protein genes are differentially expressed during the seed to seedling transition.
**Fig. S6** Dataset comparison of the translational shift genes during the seed to seedling transition.
**Fig. S7** Comparison of sequence features between genes regulated at the hydration and germination translational shift and the background.
**Fig. S8** Spatial distribution of enriched motifs in genes translationally regulated during the Arabidopsis seed to seedling transition.

**Table S1a** qRT‐PCR data for gene expression analysis and primers used
**Table S1b** Total gene set with fold change (FC), adjusted P‐value, for total RNA, polysomal RNA and polysomal occupancy changes across seed germination
**Table S1c** Comparison of the transcriptional difference in the current data set and dataset from Silva *et al*. (2016)
**Table S1d** Translational regulated genes during seed germination
**Table S1e** Dominant gene groups among the translational regulated shift genes
**Table S1f** Gene set enrichment analysis for the translational shift genes
**Table S1g** Sequence feature analysis for the translational shifts during seed germination
**Table S1h** Literature derived sequence feature analysis for the translational shifts during seed germination
**Table S1i** Ribosomal protein gene transcriptional profile during seed germination
