# Supplementary material for: Extensive translational regulation during seed germination revealed by polysomal profiling
Source: New Phytol. 2016 Dec 9;214(1):233–44. doi: 10.1111/nph.14355 (PMC5347915; doi:10.1111/nph.14355)
Supplement: Supplementary file 1 — Fig. S1 Gene 1.1 ST GeneChip quality assessment and reproducibility. Fig. S2 Coordinated transcriptional and translational expression changes during the seed to seedling transition. Fig. S3 Arabidopsis seed to seedling transition is characterized by two translational shifts. Fig. S4 Temporal functional changes of total RNA and polysomal RNA during the seed to seedling transition using over‐representation analysis. Fig. S5 Ribosomal protein genes are differentially expressed during the seed to seedling transition. Fig. S6 Dataset comparison of the translational shift genes during the seed to seedling transition. Fig. S7 Comparison of sequence features between genes regulated at the hydration and germination translational shift and the background. Fig. S8 Spatial distribution of enriched motifs in genes translationally regulated during the Arabidopsis seed to seedling transition. [file NPH-214-233-s001.pdf]

Supporting Information

Extensive Translational Regulation During Seed Germination Revealed by Polysomal Profiling

Bing Bai, Alessia Peviani, Sjors van der Horst, Magdalena Gamm, Berend Snel, Leónie Bentsink and Johannes Hanson

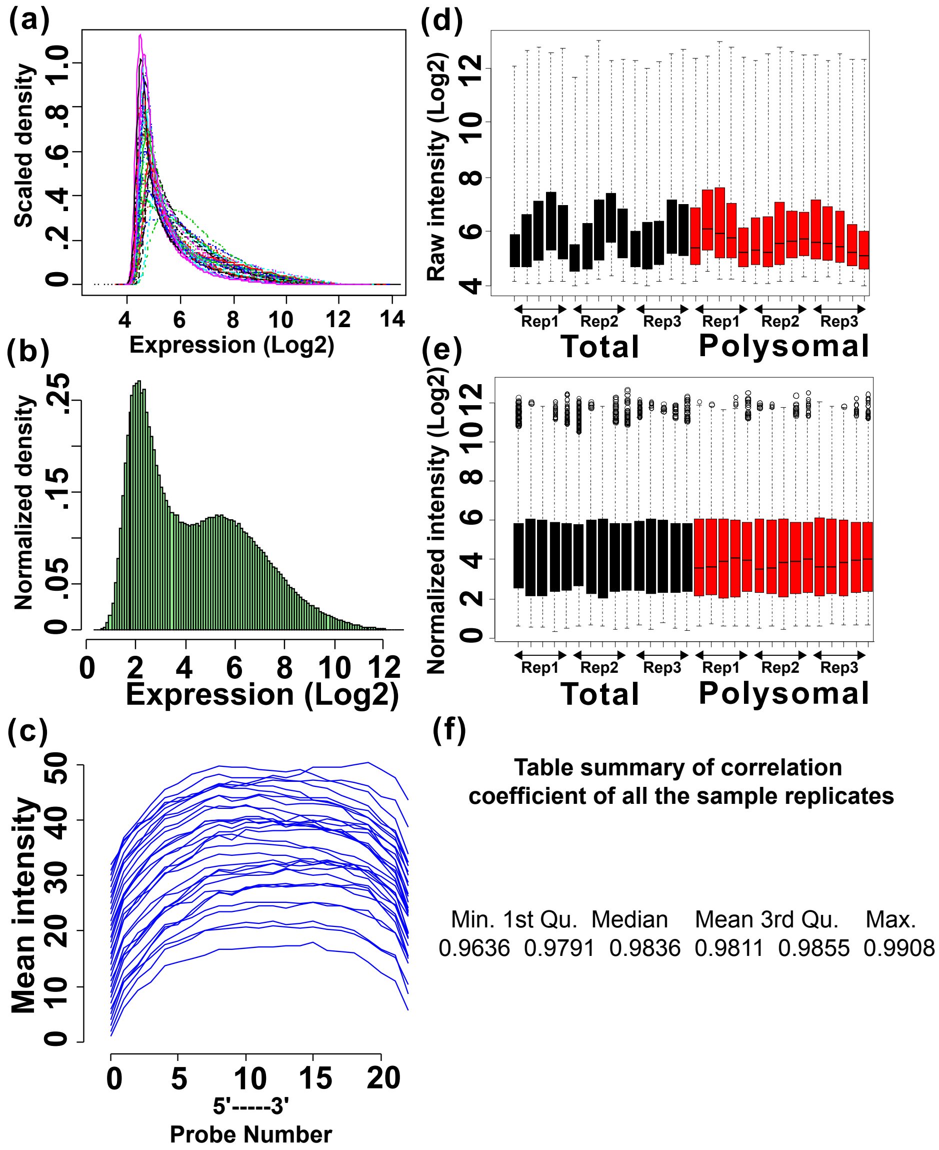

Fig. S1. Gene 1.1 ST GeneChip quality assessment and reproducibility.

(a) All 30 Gene 1.1 ST GeneChip arrays showed similar patterns of raw probe intensity. Slide images were manually inspected, with no noticeable spatial artifacts. (b) The histogram of the normalized data shows separated peaks for noise and signal, and the plot indicates a value of 4 (on a log2 scale) as being potentially expressed. (c) RNA degradation plot shows comparable slopes for all arrays. (d, e) Before and after RMA normalization the data distributions become comparable, although lower median values are found for the dry seeds sample both from total and polysomal RNA, which are samples that were isolated from metabolically less active material. (f) A summary (Minimum, 1st quartile, median, 3rd quartile and maximum value) of the correlation coefficient of all biological replicates and the correlation coefficients between all the biological replicates range from 0.96-0.99 with the average correlation coefficient of 0.98.

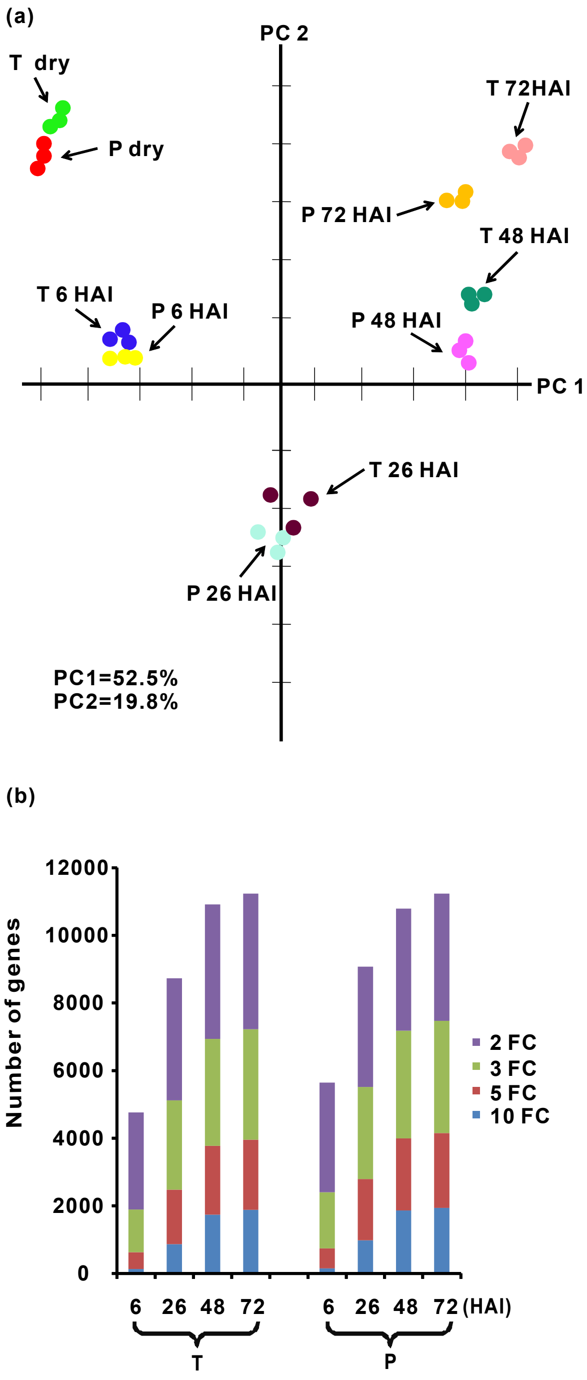

Fig. S2. Transcriptional and translational expression changes during seed to seedling transition

(a) PCA of the total RNA and polysomal RNA changes across the dry seed to seedling time course. The three replicates of total (T) and polysomal mRNA (P) are indicated by colour. The variance explained by the first two principal components is shown. (b) Transcriptional and translational differences are represented by the number of differentially expressed genes at four time points during imbibition. The bars show the number of differentially expressed genes compared with dry seeds at a 2-, 3-, 5-, and 10-fold change (FC) cut-off ($P<0.05$, FDR).

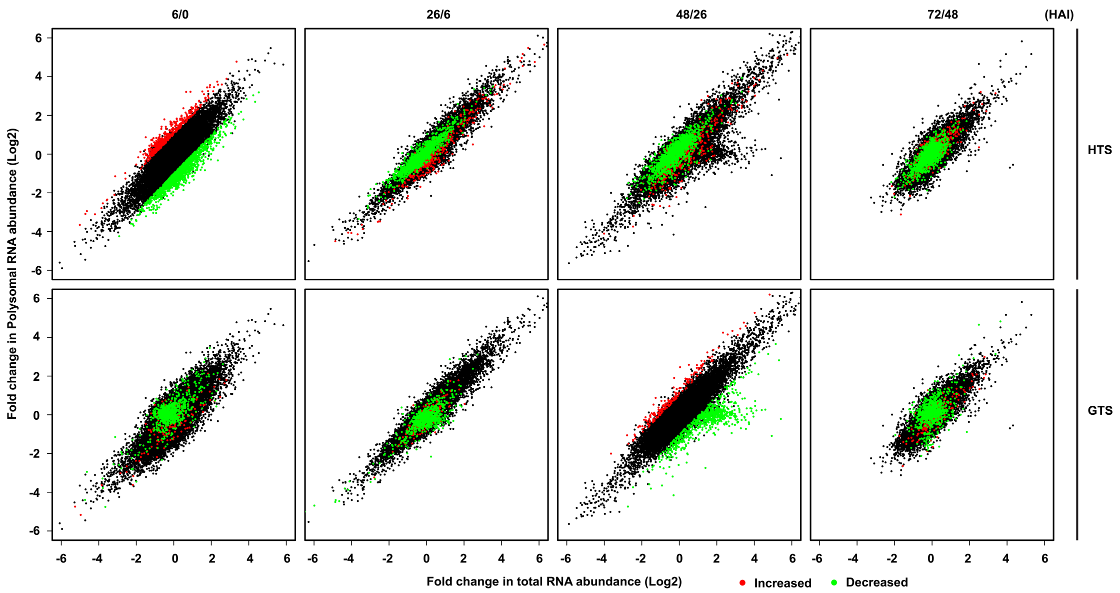

Fig. S3. Arabidopsis seed to seedling transition is characterized by two translational shifts

At large are the changes in the total mRNA and the polysomal mRNA correlated, with the exception of the genes affected by the two translational shifts indicated in colour. Genes identified with increased (red dots) or decreased (green dots) polysome occupancy are indicated for both the Hydration Translational Shift (HTS, upper panels) and the Germination Translational Shift (GTS, lower panels). The Log2 fold change of total RNA (X-axis) and polysomal RNAs (Y-axis) in the consecutive stages following seed imbibition (the comparison of 6 to 0, 26 to 6, 48 to 26 and 72 to 48 hours) after the start of imbibition (HAI) are plotted (black).

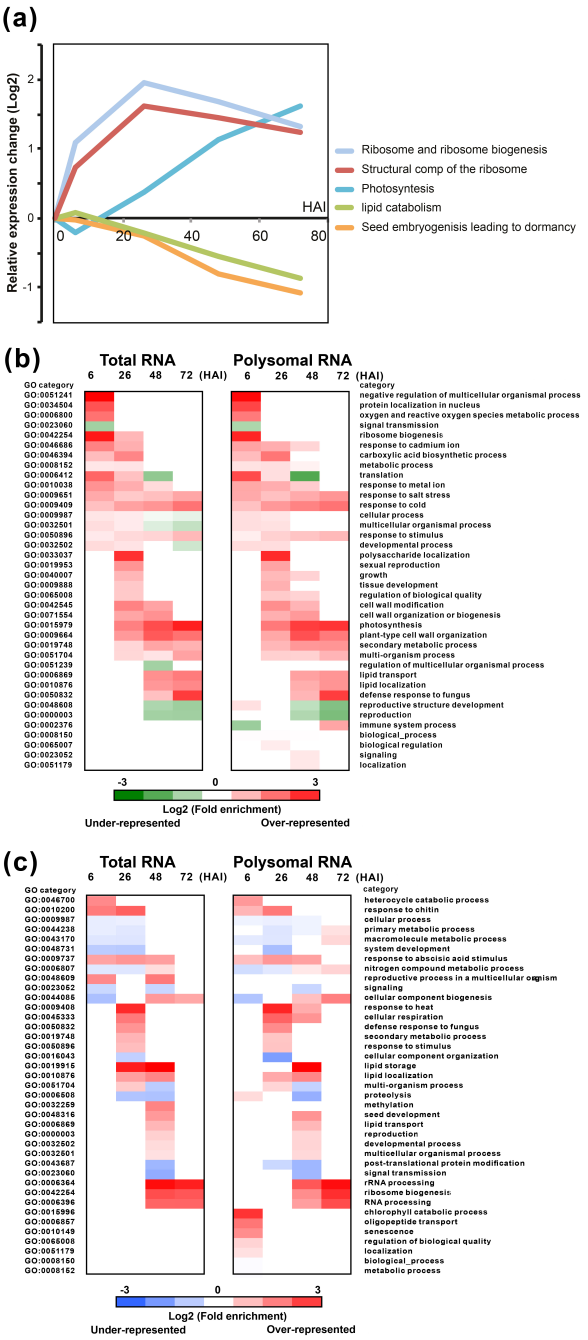

Fig. S4. Temporal functional changes of total RNA and polysomal RNA during seed to seedling transition using over-representation analysis (ORA)

(a) Relative expression change during seedling establishment of five general gene categories. The transcriptional changes of all genes belonging to the GO Category indicated were averaged and the expression value plotted is the log2 transformed compared to dry seeds at each time point. (b) The over- and under-represented gene categories of the up-regulated genes (red and green) and (c) down-regulated genes (red and blue) of the germination time course (the consecutive time points are compared) are identified in the total RNA and polysomal RNA changes by gene trails followed by REVIGO for redundancy reduction. Selected categories are summarized in the graphs, and the brightness of the red (over-represented genes) and green (b) and blue (c) (under-represented genes) shows the Log2 fold enrichment of respective category at each time point during germination.

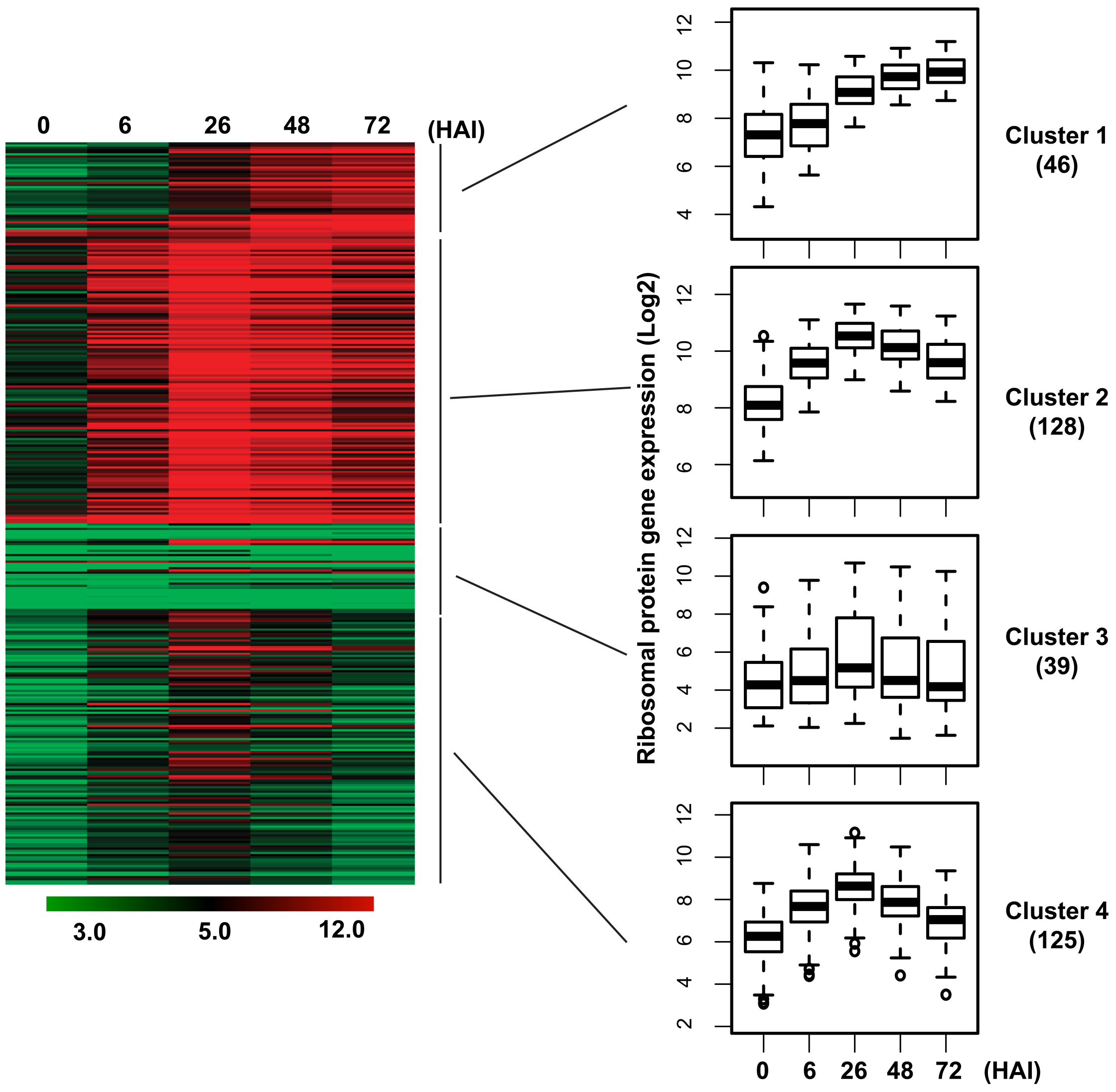

Fig. S5 Ribosomal protein genes are differentially expressed during the seed to seedling transition

Ribosomal genes (including nuclear encoded, chloroplastic and mitochondrial ribosomal proteins) detected during seed to seedling transition are clustered based on their transcriptional profile during seed germination. The heat-map indicate the expression level of the genes ordered according to the four clusters detected based on BIC (Bayesian information criterion) in a model based cluster analysis method (Lebret *et al.*, 2015). The boxplot indicates the average expression of each cluster during seed germination. The number of ribosomal protein genes of each cluster is indicated, within brackets) and provided in Table S1I.

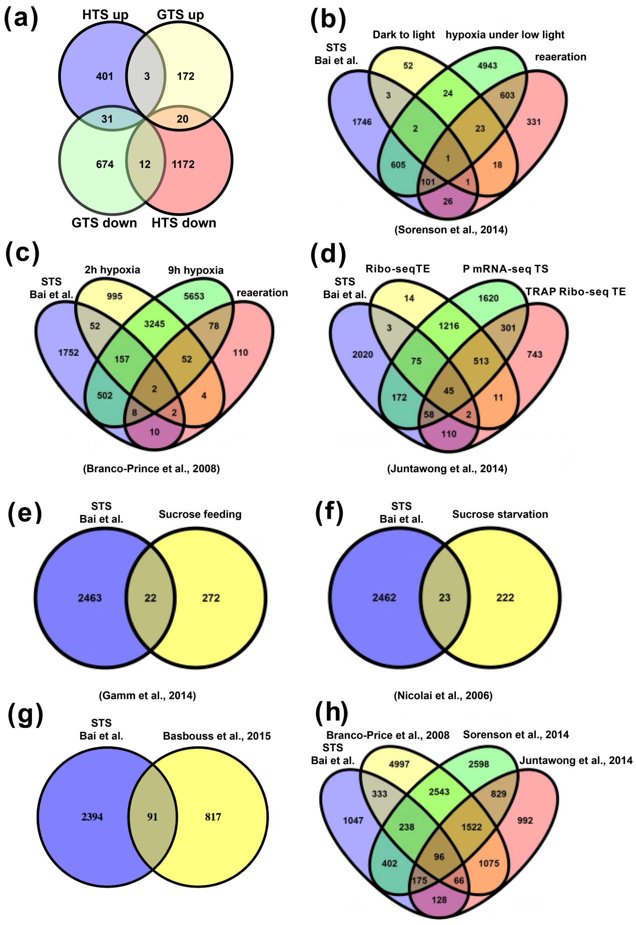

Fig. S6. Dataset comparison of the translational shift genes during seed to seedling transition

Two translational shifts (Hydration Translational Shift, HTS and Germination Translational Shift, GTS) during seed germination showed distinct translational selectivity. (b-g) Data set comparison for the gene between the seed translational shifts (HTS + GTS) from current research and genes under translational control in different environmental and developmental cues. (b) Seed Translational Shift (STS) in the current study (Bai et al.) compared with genes under translational control from dark to light (DL), treated with 2 h hypoxia under low light followed by 20 min reaeration as (Sorenson & Bailey-Serres, 2014). (c) STS compared with gene translational regulated under 2 h and 9 h oxygen and carbon dioxide deprivation and following 1 h re-aeration as (Branco-Price *et al.*, 2008) (d) STS compared with genes changed in translational efficiency evaluated by ribosome footprint (RF) sequencing (Ribo-seq TE) and translating ribosome affinity purification based RF sequencing (TRAP-Ribo-seq TE) and also genes changed in translational status evaluated by translation status defined by comparing polysomal mRNA to total mRNA abundance (P mRNA-seq TS) during 2h hypoxia as (Juntawong *et al.*, 2014) (e) STS compared with genes translationally regulated by sucrose feeding as (Gamm *et al.*, 2014) (f) STS compared with gene translationally affected by sucrose starvation of Arabidopsis cell culture as (Nicolai *et al.*, 2006). (g) The comparison of STS with genes changed in polyosomal RNA abundance affected by seed dormancy during seed imbibition (Basbouss-Serhal *et al.*, 2015) (Note the data from supplementary table is used, which is deviating from the numbers of regulated genes given in the text of the main manuscript). (h) The comparison between the STS in the current study with all three translationally regulated gene sets from (b), (c) and (d).

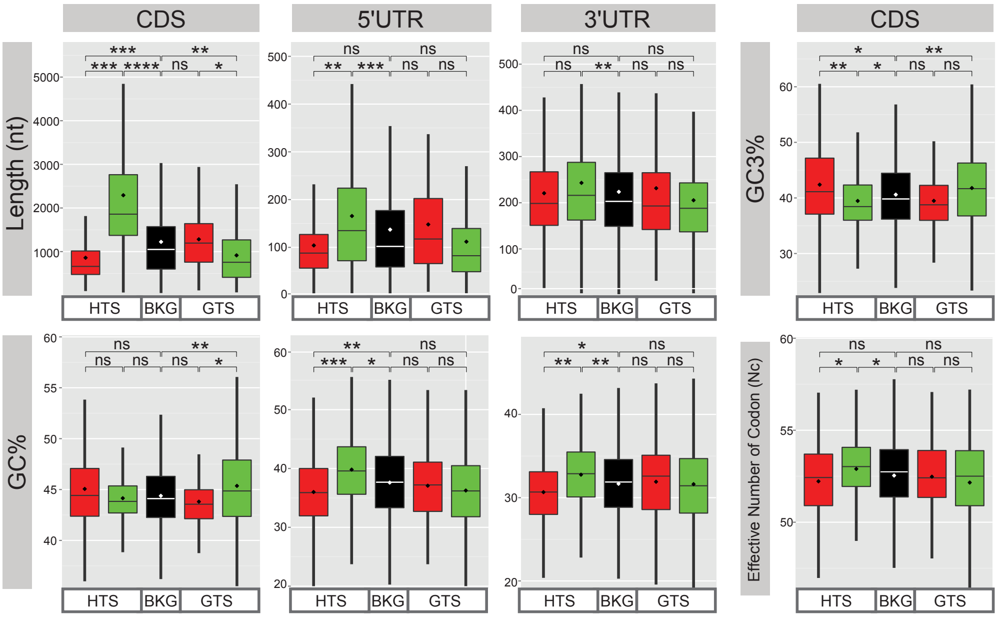

Fig. S7. Comparison of sequence features between genes regulated at the Hydration and Germination Translational Shift and the background

Length and GC content comparison across coding sequence (CDS), 5'UTR, 3'UTR (TAIR10) and GC3 content and codon bias (Nc) comparison across CDS of Hydration Translational Shift genes (HTS) and Germination Translational Shift genes (GTS) and microarray background (BKG). Colour scheme: microarray background (black), translationally up- (red) or down-regulated (blue) genes. *P*-value<= E-5 (*), E-10 (**), <= E-20 (***), <= E-50 (****); p-values higher than E-5 are considered not significant (ns). P-values refer to the difference in median, as calculated using a 1-tailed Wilcoxon test. Diamonds in the boxplots indicate the mean of the distribution; outliers (more than 1.5 IQR lower than the 1st or higher than the 3rd quartile) were excluded to improve the visualization of the results.

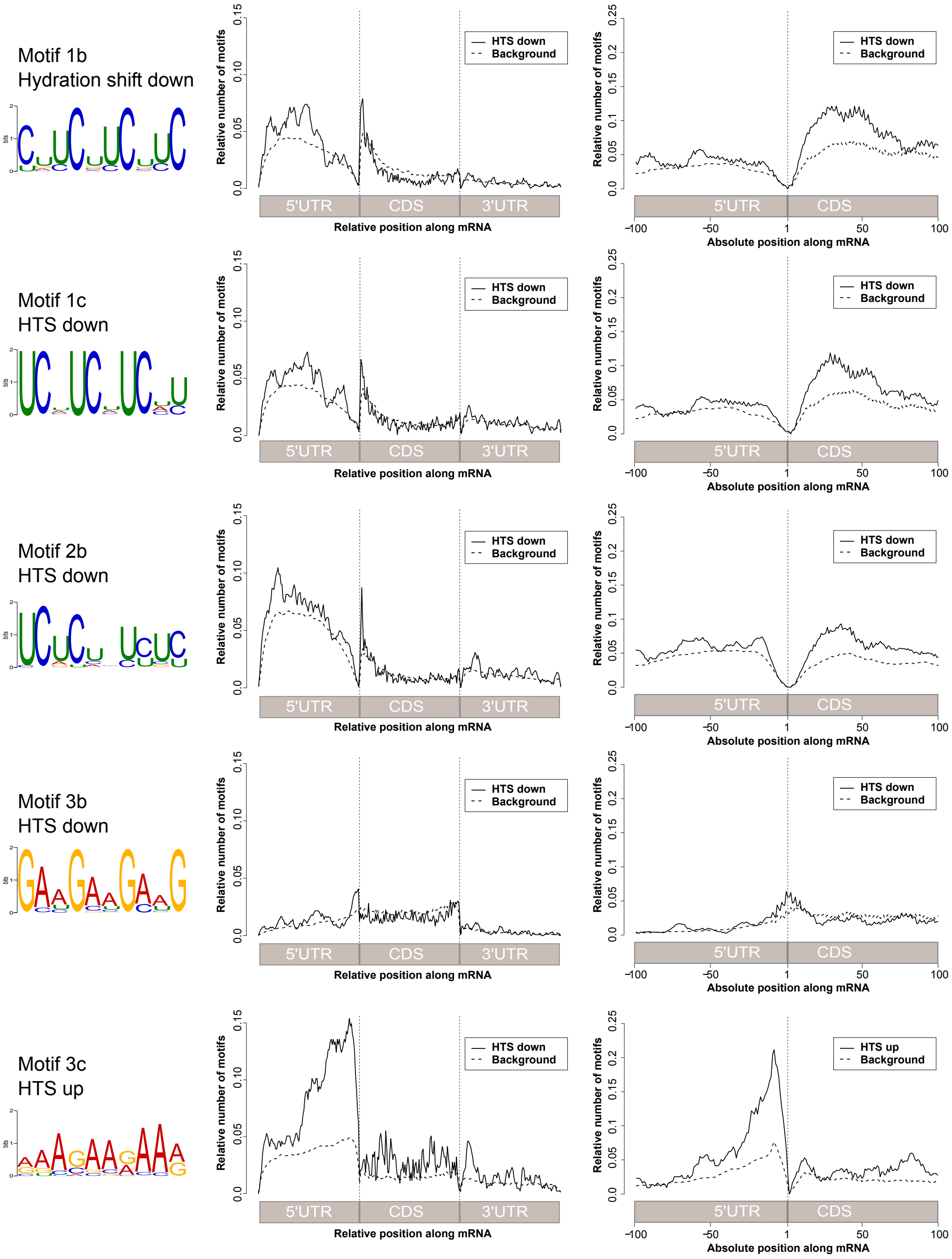

Fig. S8. Spatial distribution of enriched motifs

Three over-represented motifs of HTS repressed transcripts (motif 1b, 1c, 2b and 3b) and one over-represented motif of HTS enhanced transcripts (motif 3c), identified by motif finder MEME. The consensus sequences are illustrated and the relative number of motifs plotted along the 5'UTR, CDS and 3'UTR in both relative and absolute manners.

Table S1. (Separate file)

a qRT-PCR data for gene expression analysis and primer used. *Spikein-normalized qPCR data for relative quantification of ribosomal RNAs in different organelles and primers used for ribosomal RNA quantification and poly (A) spikeins are listed.

b Total gene set with fold change (FC, adjusted P-value,) for total RNA, polysomal RNA and translational efficiency changes across seed germination.

c Comparison of the transcriptional difference in the current data set and dataset from(Silva *et al.*, 2016) *TR: Testa rupture; GC: Greening cotyledon; OC: Opening cotyledon

d Translational regulated genes during seed germination. *Genes are listed as gene model, gene annotation, shift type during seed germination subgroup, Polysomal occupancy (PO), intensity, log2(fold change), adjusted p value and the indication of the significance.

e Dominant gene groups among the translational regulated shift genes.*Genes are listed as gene model, gene annotation, shift type during seed germination subgroup, Polysomal occupancy (PO), intensity, log2(fold change), adjusted p value and the indication of the significance.

f Gene set enrichment analysis for the translational shift genes. *GO-terms are listed by their shift type, GO term ID, GO description, significance of the enrichment, FDR, genes in each GO category.

g Sequence feature analysis for the translational shifts during seed germination

h Literature derived sequence feature analysis for the translational shifts during seed germination. *The comparisons are made between the up- and down-regulated shifts as well as each shift to the background (BKG) respectively. The compared sequence features include the rate of decay (k decay), the 95% confidence interval for the rate of decay (k 97.5%, k 2.5%), the standard error for the rate of decay, the mRNA half-life (t1/2, hours), the t value associated with the regression fit (tValue), the probability associated with the t-value for the regression (Probt), the number of introns, the length and the AU% in the 3' UTR (3p_), the coding sequence (cds_), 5' UTR (5p_) and the whole mRNA for each transcript (Narsai *et al.*, 2007). The column from left to right indicate the mean value of each compared group, p value for the t-test indicating the significance ($p<0.05$), t statistics and the confidence intervals.

I Ribosomal protein gene transcriptional profile during seed germination.

References

Basbouss-Serhal I, Soubigou-Taconnat L, Bailly C, Leymarie J. **2015**. Germination Potential of Dormant and Nondormant Arabidopsis Seeds Is Driven by Distinct Recruitment of Messenger RNAs to Polysomes. *Plant Physiology* **168**: 1049–1065.

Branco-Price C, Kaiser KA, Jang CJH, Larive CK, Bailey-Serres J. **2008**. Selective mRNA translation coordinates energetic and metabolic adjustments to cellular oxygen deprivation and reoxygenation in *Arabidopsis thaliana*. *The Plant Journal* **56**: 743–755.

Gamm M, Peviani A, Honsel A, Snel B, Smeekens SCM, Hanson J. **2014**. Increased sucrose levels mediate selective mRNA translation in Arabidopsis. *BMC Plant Biol* **14**: 306.

Juntawong P, Girke T, Bazin J, Bailey-Serres J. **2014**. Translational dynamics revealed by genome-wide profiling of ribosome footprints in Arabidopsis. *Proceedings of the National Academy of Sciences of the United States of America* **111**: E203–12.

Lebret R, Iovleff S, Langrognet F, Biernacki C, Celeux G, Govaert G. 2015. Rmixmod: The R Package of the Model-Based Unsupervised, Supervised, and Semi-Supervised Classification Mixmod Library. Journal of Statistical Software 67(6): 1-29.

Narsai R, Howell KA, Millar AH, O'Toole N, Small I, Whelan J. **2007**. Genome-wide analysis of mRNA decay rates and their determinants in *Arabidopsis thaliana*. *Plant Cell* **19**: 3418–3436.

Nicolai M, Roncato MA, Canoy AS, Rouquie D, Sarda X, Freyssinet G, Robaglia C. **2006**. Large-scale analysis of mRNA translation states during sucrose starvation in arabidopsis cells identifies cell proliferation and chromatin structure as targets of translational control. *Plant Physiology* **141**: 663–673.

Silva AT, Ribone PA, Chan RL, Ligterink W, Hilhorst HWM. **2016**. A Predictive Coexpression Network Identifies Novel Genes Controlling the Seed-to-Seedling Phase Transition in Arabidopsis thaliana. *Plant Physiology* **170**: 2218–2231.

Sorenson R, Bailey-Serres J. **2014**. Selective mRNA sequestration by OLIGOURIDYLATE-BINDING PROTEIN 1 contributes to translational control during hypoxia in Arabidopsis. *Proceedings of the National Academy of Sciences of the United States of America* **111**: 2373–2378.
